# Prevalence and Severity of Sesame Allergy in the United States

**DOI:** 10.1001/jamanetworkopen.2019.9144

**Published:** 2019-08-02

**Authors:** Christopher M. Warren, Avneet S. Chadha, Scott H. Sicherer, Jialing Jiang, Ruchi S. Gupta

**Affiliations:** 1 Center for Food Allergy and Asthma Research, Northwestern University Feinberg School of Medicine and Ann & Robert H Lurie Children’s Hospital of Chicago, Chicago, Illinois; 2 Elliot and Roslyn Jaffe Food Allergy Institute, Division of Allergy, Department of Pediatrics, Mount Sinai School of Medicine, New York, New York

## Abstract

**Importance:**

Sesame allergy is of growing concern in the United States. The US Food and Drug Administration recently issued a request for epidemiological data on the prevalence and severity of sesame allergies in the United States to inform possible regulatory action requiring sesame to be labeled as an allergen on packaged foods.

**Objective:**

To provide current estimates of the prevalence, severity, distribution, and clinical characteristics of sesame allergy in the United States.

**Design, Setting, and Participants:**

This cross-sectional study assessed findings of web- and telephone-based food allergy questionnaires. Study participants were first recruited from NORC (National Opinion Research Center) at the University of Chicago’s probability-based AmeriSpeak panel, and additional participants were recruited through Survey Sampling International. Surveys were administered to a nationally representative sample of 51 819 US households from October 1, 2015, through September 31, 2016. Responses for 40 453 adults and 38 408 children were included in the analysis. Data were analyzed from January 1, 2017, through May 1, 2019.

**Exposures:**

Demographic and allergic characteristics of participants.

**Main Outcomes and Measures:**

Self-reported sesame allergy was the main outcome and was considered convincing if reported symptoms to sesame-allergic reactions were consistent with an IgE-mediated reaction. Diagnostic history of specific allergens and use of food allergy–related health care services were also primary outcomes.

**Results:**

Using survey responses from 78 851 individuals, an estimated 0.49% (95% CI, 0.40%-0.58%) of the US population reported a current sesame allergy, whereas 0.23% (95% CI, 0.19%-0.28%) met symptom-report criteria for convincing IgE-mediated allergy. An additional 0.11% (95% CI, 0.08%-0.16%) had a sesame allergy reported as physician diagnosed but did not report reactions fulfilling survey-specified convincing reaction symptoms. Among individuals with convincing IgE-mediated sesame allergy, an estimated 23.6% (95% CI, 16.9%-32.0%) to 37.2% (95% CI, 29.2%-45.9%) had previously experienced a severe sesame-allergic reaction, depending on the definition used, and 81.6% (95% CI, 71.0%-88.9%) of patients with convincing sesame allergy had at least 1 additional convincing food allergy. Roughly one-third of patients with convincing sesame allergy (33.7%; 95% CI, 26.3%-42.0%) reported previous epinephrine use for sesame allergy treatment.

**Conclusions and Relevance:**

These data indicate a substantial burden of sesame allergy and provide valuable context to physicians and policy makers in their efforts to evaluate and reduce the public health burden of sesame allergy. Moreover, the relatively low rates of physician diagnosis observed among individuals with convincing IgE-mediated sesame allergy seem to indicate the need for clear diagnosis and appropriate management.

## Key Points

**Question:**

What is the current population-level burden of sesame allergy in the United States?

**Findings:**

Using a cross-sectional population-based survey, an estimated 0.49% of the US population reported a current sesame allergy, whereas 0.23% of the population had a convincing sesame allergy using confirmatory symptom report criteria.

**Meaning:**

The findings suggest that sesame allergy is a relatively common, frequently comorbid food allergy that affects patients of all ages, resulting in severe reactions and health care utilization.

## Teaser

This cross-sectional, population-based study provides current estimates of the prevalence, severity, distribution, and clinical characteristics of sesame allergy in US adults and children.

## Introduction

Food allergy is a serious health issue in the United States, reportedly affecting approximately 8% of children^1^ and 10% of adults,^2^ resulting in substantial psychosocial,^3^ economic,^4^ and physical health^5^ burdens. Although the 8 most common types of food allergies in the United States have been established for some time,^6^ given the growing number of people affected by food allergies across the United States, it is important to look beyond the top 8 to other allergies that may also have a substantial population-level effect. One allergen of growing concern is sesame, which is a regulated food allergen in many countries^7^ and was recently estimated to affect 0.23% of US children and adults.^1,2^ A smaller, population-based survey conducted more than a decade ago also identified sesame as a food allergy of concern, affecting approximately 0.1% of the US children and adults.^8^ Previous work also suggests that sesame allergy can persist into adulthood and result in severe reactions, including anaphylaxis.^9^

Better understanding the US population-level effect of sesame allergy is critical, owing to the fact that the US Food and Drug Administration mandates the labeling of only 8 allergenic foods and/or food groups: peanut, milk, shellfish, tree nuts, egg, soy, fin fish, and wheat. Currently, sesame may escape labeling legally by being included under nonspecific terms such as *flavors* or may be referred to by words that may not be easily recognized by consumers as containing sesame, such as *tahini*. The decision to not include sesame in labeling laws was made when the aforementioned 8 allergens were believed to be responsible for 90% of food allergies.^10^ The lack of current data on sesame allergy recently led the US Food and Drug Administration to issue a request in 2018 for information regarding “the prevalence and severity of sesame allergies in the United States to inform possible regulatory action that would require sesame to be labeled as an allergen on packaged foods.”^11^ Given the need for data on sesame allergy among US children and adults, this study reports current estimates of the prevalence, severity, symptoms, distribution, use of health care services, and determinants of sesame allergy.

## Methods

A detailed food allergy prevalence survey was administered to a nationally representative sample of US households from October 1, 2015, through September 30, 2016. The institutional review boards of at the University of Chicago and Northwestern University approved the study protocol and approved all study activities. Informed consent (written and oral) was obtained from all participants. This study followed the Consolidated Standards of Reporting Trials (CONSORT) reporting guideline.

### Survey Development and Design

The original 2009-2010 survey was developed by pediatricians, pediatric allergists, and health services researchers with support of an expert panel consisting of leaders in the field. Expert panel review and key informant cognitive interviews were conducted using the approach described by Gupta et al^12^ to ensure survey functionality as well as understandability and consistency of responses. To promote comparability of estimates between survey administrations, the core elements of the survey were kept intact. Additional constructs were added to the 2015-2016 instrument to assess emerging research questions relating to the etiology and management of food allergy. The final survey was administered electronically via the web or telephone. Additional details are described by Gupta et al.^1^

### Study Participants, Recruitment, and Survey Weighting

Eligible study participants included adults (≥18 years old) able to complete the survey in English or Spanish via the web or telephone and who resided in a US household. As in the 2009-2010 survey, this study relied on a nationally representative household panel to support population-level inference.^6^ Study participants were first recruited from NORC at the University of Chicago probability-based AmeriSpeak panel, with 7218 responses of 14 095 invitees resulting in a survey completion rate of 51.2%. Each child and adult participant was assigned a base, study-specific sampling weight equal to their or their responding parent’s nonresponse-adjusted AmeriSpeak sampling weight. Iterative proportional fitting methods were applied to rake adult sampling weights to external population totals associated with age, sex, educational attainment, race/ethnicity, housing tenure, telephone status, and Census Division to improve external validity. Child-specific weights were further adjusted to account for random selection of as many as 3 children per household and raked to external pediatric US population totals.

To increase precision and ensure sufficient sample size among key subpopulations, prevalence estimates gleaned from population-weighted AmeriSpeak responses were augmented by calibration-weighted, nonprobability-based responses obtained through Survey Sampling International. The final, combined sample weight was derived by applying an optimal composition factor that minimizes mean square error associated with food allergy prevalence estimates. Gupta et al^1^ provide detailed information regarding the complex survey sampling, weighting, and analysis methods used. In total, surveys were completed by 51 819 US households from all 50 states and the District of Columbia, providing parent-proxy responses for 38 408 children and self-report for 40 443 adults. Respondents received $5 on survey completion.

### Outcome Measures

Reported food allergies, including sesame, were considered convincingly IgE mediated (hereinafter termed *convincing*) if the most severe reaction ever reported to that food included at least 1 symptom on the stringent symptom list developed by our expert panel (eFigure 1 in the Supplement). Reported allergies with reaction symptoms characteristic of oral allergy syndrome or food intolerances were excluded from categorization as convincing per the food allergy categorization flowchart summarized in eFigure 2 in the Supplement, even if such allergies were physician diagnosed. Convincing food allergies for which parents reported a physician’s diagnosis were considered physician-confirmed food allergies. For each convincing allergy, a severe reaction history was indicated by the presence of multiple stringent symptoms occurring within 2 or more of the following 4 organ systems: skin/oral mucosa, gastrointestinal tract, cardiovascular, and respiratory tract. An alternative definition of severe reaction symptoms used in some prior work^13^ was also applied, which included only individuals reporting sesame-induced wheeze, fainting or dizziness, and/or low blood pressure.

### Statistical Analysis

Data were analyzed from January 1, 2017, through May 1, 2019. Complex survey-weighted proportions and 95% CIs were calculated to estimate prevalence, and robust standard errors accounted for household-level clustering. Data analysis was conducted using Stata statistical software, version 14 (StataCorp). Weighted Pearson χ^2^ statistics, which were corrected for the complex survey design with the second-order correction of Rao and Scott^14^ and converted into *F* statistics, were used for all comparisons of sesame allergy and participant characteristics. Two-sided *P* < .05 was indicative of statistical significance.

## Results

Among the final sample of 78 851 individuals, 251 respondents (102 children and 149 adults) were categorized as having convincing sesame allergy using criteria that required experiencing at least 1 stringent symptom. These data correspond to an estimated 0.23% (95% CI, 0.19%-0.28%) of the US general population. An additional 0.11% (95% CI, 0.08%-0.16%) had a sesame allergy reported as physician diagnosed but did not report reactions fulfilling survey-specified convincing reaction symptoms. Among children, 0.21% (95% CI, 0.16%-0.28%) were estimated to have convincing sesame allergy compared with 0.24% (95% CI, 0.19%-0.29%) of adults (Table 1). Nearly twice as many children (n = 206) and adults (n = 273) reported that they were allergic to sesame (0.49%; 95% CI, 0.40%-0.58); however, half of these individuals reported reaction symptoms that did not meet established criteria^1,2^ for convincing, IgE-mediated food allergy classification and therefore were not included in overall, convincing food allergy estimates. In addition, only 55.5% (95% CI, 41.6%-68.6%) of children and 37.7% (95% CI, 28.7%-47.6%) of adults with convincing IgE-mediated sesame allergy had a physician diagnosis for sesame allergy. Conversely, a substantial proportion (41.3%; 95% CI, 33.2%-49.9%) of individuals reporting a physician-diagnosed sesame allergy did not meet our symptom report criteria for a convincing IgE-mediated sesame allergy.

**Table 1.** Prevalence of Sesame Allergies by Age and Frequency

Age Group	Sesame Allergy Characteristic			
	Reported	History of Convincing Symptoms or Physician Diagnosis	History of Convincing Symptoms	Physician Diagnosis
No. of children	206	158	102	56
No. of adults	273	204	149	61
**Prevalence**^a^				
All	0.49 (0.40-0.58)	0.34 (0.29-0.41)	0.23 (0.19-0.28)	0.10 (0.08-0.12)
All children (<18 y)	0.53 (0.39-0.72)	0.41 (0.28-0.60)	0.21 (0.16-0.28)	0.12 (0.08-0.17)
0-2 y	0.58 (0.34-0.99)	0.38 (0.22-0.68)	0.26 (0.12-0.54)	0.12 (0.04-0.39)
3-5 y	0.45 (0.29-0.70)	0.34 (0.21-0.56)	0.23 (0.13-0.41)	0.16 (0.08-0.32)
6-10 y	0.61 (0.36-1.02)	0.53 (0.29-0.95)	0.27 (0.16-0.44)	0.15 (0.08-0.30)
11-13 y	0.74 (0.28-1.95)	0.61 (0.19-2.0)	0.14 (0.07-0.27)	0.09 (0.04-0.21)
14-17 y	0.31 (0.17-0.55)	0.20 (0.13-0.32)	0.15 (0.09-0.26)	0.06 (0.03-0.11)
All adults (≥18 y)	0.44 (0.38-0.52)	0.32 (0.27-0.39)	0.24 (0.19-0.29)	0.09 (0.07-0.12)
18-29 y	0.59 (0.46-0.76)	0.47 (0.36-0.61)	0.33 (0.24-0.44)	0.17 (0.11-0.26)
30-39 y	0.62 (0.49-0.81)	0.44 (0.32-.060)	0.31 (0.21-0.45)	0.12 (0.07-0.21)
40-49 y	0.53 (0.36-0.77)	0.31 (0.20-0.46)	0.23 (0.15-0.37)	0.08 (0.04-0.16)
50-59 y	0.42 (0.26-0.67)	0.37 (0.22-0.62)	0.28 (0.15-.053)	0.10 (0.05-0.21)
≥60 y	0.17 (0.10-0.29)	0.12 (0.07-0.22)	0.09 (0.04-0.17)	0.003 (0.00-0.02)
**Prevalence With Any Convincing Food Allergy**^a^				
All	4.18 (3.44-5.07)	2.91 (2.44-3.47)	2.29 (1.92-2.74)	0.95 (0.75-1.19)
All children (<18 y)	5.4 (3.9-7.5)	4.3 (2.9-6.4)	2.7 (2.1-3.6)	1.5 (1.0-2.2)
0-2 y	6.31 (3.66-10.66)	4.9 (2.6-8.9)	3.55 (1.68-7.38)	1.67 (0.51-5.29)
3-5 y	4.02 (2.41-6.62)	3.7 (2.2-6.3)	2.74 (1.50-4.97)	1.86 (0.90-3.83)
6-10 y	5.14 (3.50-7.49)	4.3 (2.8-6.6)	3.33 (2.00-5.49)	1.94 (0.99-3.75)
11-13 y	8.46 (2.93-22.08)	6.7 (1.7-22.6)	1.83 (0.95-3.52)	1.24 (0.55-2.76)
14-17 y	4.09 (2.23-7.41)	2.7 (1.7-4.2)	2.11 (1.20-3.67)	0.82 (0.42-1.58)
All adults (≥18 y)	3.3 (2.8-3.9)	2.6 (2.2-3.2)	2.20 (1.8-2.7)	0.82 (0.62-1.1)
18-29 y	4.28 (3.28-5.56)	3.4 (2.6-4.5)	2.90 (2.14-3.92)	1.47 (0.93-2.31)
30-39 y	4.22 (3.21-5.53)	3.0 (2.1-4.1)	2.44 (1.66-3.56)	0.97 (0.57-1.67)
40-49 y	3.64 (2.53-5.22)	2.8 (1.9-4.2)	2.33 (1.49-3.62)	0.81 (0.40-1.63)
50-59 y	3.32 (2.04-5.37)	2.9 (1.7-5.0)	2.38 (1.27-4.42)	0.84 (0.41-1.74)
≥60 y	1.25 (0.70-2.20)	1.1 (0.6-2.0)	1.00 (0.51-1.93)	0.03 (0.004-0.21)

^a^ Expressed as frequency (95% CI) in percentages.

### Demographics

Detailed demographic characteristics of individuals with convincing sesame allergy (hereinafter termed *sesame allergy* unless otherwise specified) are listed in Table 2. Of these, 55.2% (95% CI, 46.9%-63.3%) were female, compared with 51.1% (95% CI, 50.5%-51.6%; *P* = .33) of the general US population. Just more than half (56.2%; 95% CI, 47.2%-64.8%) were white, a slightly lower proportion than the general US population (62.2%; 95% CI, 61.4%-62.9%; *P* = .17), whereas 24.7% (95% CI, 17.6%-33.6%) were Hispanic, significantly exceeding the proportion of Hispanic individuals in the US population (17.4%; 95% CI, 16.7%-18.1%; *P* = .04) and greater than the proportion of Hispanic individuals among those with the top 8 most prevalent food allergies (19.4%; 95% CI, 17.8%-21.2%). No significant differences in household income were found among individuals with sesame allergy. Although overall sesame allergy prevalence rates differed among the 4 census regions, ranging from 0.19% (95% CI, 0.12%-0.28%) in the Midwest to 0.37% (95% CI, 0.24%-0.56%) in the Northeast, sesame allergy was estimated to affect residents of all 50 states.

**Table 2.** Demographic Distribution of Convincing Sesame Allergy vs No Allergy and Top 8 Allergies

Variable	Population-Weighted Frequency, % (95% CI)			Current Convincing Allergy to Other Top 8, Population-Weighted Frequency (95% CI), %	*P* Value
	All Respondents	Current Convincing Sesame Allergy	*P* Value		
Race/ethnicity					
Asian non-Hispanic	3.7 (3.5-4.0)	3.0 (1.3-7.0)	.63	4.3 (3.6-5.0)	.43
Black non-Hispanic	12.0 (11.6-12.5)	13.1 (8.5-19.4)	.70	13.6 (12.3-15.1)	.84
White non-Hispanic	62.2 (61.4-62.9)	56.2 (47.2-64.8)	.17	56.8 (54.8-58.7)	.90
Hispanic	17.4 (16.7-18.1)	24.7 (17.6-33.6)	.04	19.4 (17.8-21.2)	.17
Multiple or other	4.7 (4.4-4.9)	3.0 (1.2-7.4)	.35	5.9 (5.0-7.0)	.15
Sex					
Male	48.9 (48.4-49.5)	44.8 (36.7-53.1)	.33	38.9 (37.1-40.7)	.17
Female	51.1 (50.5-51.6)	55.2 (46.9-63.3)		61.1 (59.3-62.9)	
Age, y					
0-2	3.6 (.3.4-3.8)	4.0 (1.9-8.0)	.01	3.0 (2.4-3.8)	.39
3-5	3.6 (3.5-3.8)	3.6 (2.0-6.5)		3.2 (2.7-3.8)	
6-10	6.2 (6.0-6.5)	6.2 (4.3-11.8)		5.7 (5.1-6.4)	
11-13	3.7 (3.5-3.9)	2.2 (1.2-4.3)		3.3 (2.8-3.9)	
14-17	5.2 (5.0-5.5)	3.4 (1.9-5.9)		4.0 (3.5-4.6)	
18-29	16.7 (16.2-17.2)	23.7 (17.6-31.0)		19.5 (18.0-21.2)	
30-39	13.2 (12.8-13.5)	17.7 (12.3-24.9)		17.4 (16.0-18.9)	
40-49	13.0 (12.6-13.4)	13.2 (8.5-19.8)		12.7 (11.5-13.9)	
50-59	14.0 (13.6-14.4)	17.2 (9.7-28.6)		15.5 (14.2-16.9)	
≥60	20.8 (20.3-21.3)	7.9 (4.1-14.5)		15.8 (14.5-17.3)	
Household income, $					
<25 000	16.5 (16.0-17.0)	11.9 (7.4-18.5)	.15	15.6 (14.2-17.0)	.25
25 000-49 999	22.0 (21.4-22.6)	21.6 (14.9-30.1)	.91	22.1 (20.6-23.6)	.90
50 000-99 999	31.0 (30.3-31.7)	35.1 (27.8-43.2)	.28	34.0 (32.3-35.9)	.79
100 000-149 999	19.5 (18.9-20.2)	23.1 (15.2-33.5)	.42	19.9 (18.2-21.7)	.48
≥150 000	11.0 (10.5-11.6)	8.4 (5.0-13.7)	.28	8.5 (7.4-9.7)	.97
Physician-diagnosed comorbid conditions					
Asthma	12.2 (11.8-12.7)	27.2 (20.5-35.1)	<.001	26.3 (24.7-28.0)	.82
Atopic dermatitis or eczema	6.5 (6.2-6.9)	13.7 (9.0-20.3)	<.001	13.1 (11.9-14.4)	.83
Eosinophilic esophagitis	0.2 (0.1-0.2)	3.6 (1.4-8.7)	<.001	0.5 (0.4-0.8)	<.001
Food protein–induced enterocolitis	0.3 (0.2-0.3)	4.4 (2.4-7.9)	<.001	1.8 (1.4-2.2)	.004
Environmental allergies	19.5 (19.0-20.0)	24.6 (18.1-32.6)	.13	32.9 (31.2-34.7)	.04
Insect sting allergy	3.5 (3.3-3.7)	7.0 (3.8-12.6)	.02	7.0 (6.2-7.9)	.99
Latex allergy	2.0 (1.9-2.2)	8.6 (5.1-14.1)	<.001	6.3 (5.4-7.3)	.26
Medication allergy	11.3 (11.0-11.7)	18.8 (11.3-29.6)	.048	18.5 (17.1-20.0)	.95
Urticaria or chronic hives	0.8 (0.7-0.9)	1.7 (0.6-5.0)	.15	2.3 (1.9-2.8)	.60
Other chronic condition	6.4 (6.1-6.7)	8.1 (4.6-13.8)	.40	7.9 (6.9-8.9)	.92
Parental history of atopy					
Asthma	14.7 (14.2-15.2)	41.0 (31.4-51.4)	<.001	26.6 (24.8-28.3)	.002
Eczema	11.1 (10.6-11.5)	29.9 (22.3-38.7)	<.001	19.7 (18.2-21.3)	.007
Allergic rhinitis	31.6 (30.9-32.4)	52.5 (42.9-61.9)	.001	47.5 (45.4-49.7)	.33
Food allergy	14.6 (14.1-15.1)	50.4 (40.3-60.4)	<.001	42.2 (40.1-44.3)	.12

Among children (aged <18 years) with sesame allergy, the median age of reported first reactions to sesame was 3.5 years (interquartile range, 1-5 years), whereas among adults it was 10 years (interquartile range, 5-19 years). Overall, 25.7% (95% CI, 18.1%-35.1%) of adults with sesame allergy reported an adult-onset sesame allergy.

### Comorbid Conditions

Individuals with sesame allergy were more likely to have other atopic conditions than were those without sesame allergy, including asthma (27.2% [95% CI, 20.5%-35.1%] vs 12.2% [11.8%-12.7%]; *P* < .001), eczema (13.7% [95% CI, 9.0%-20.3%] vs 6.5% [95% CI, 6.2%-6.9%]; *P* < .001), eosinophilic esophagitis (3.6% [95% CI, 1.4%-8.7%] vs 0.2% [95% CI, 0.1%-0.2%]; *P* < .001), food protein–induced enterocolitis (4.4% [95% CI, 2.4%-7.9%] vs 0.3% [95% CI, 0.2%-0.3%]; *P* < .001), insect sting allergies (7.0% [95% CI, 3.8%-12.6%] vs 3.5% [95% CI, 3.3%-3.7%]; *P* = .02), latex allergies (8.6% [95% CI, 5.1%-14.1%] vs 2.0% [95% CI, 1.9%-2.2%]; *P* < .001), and medication allergy (18.8% [95% CI, 11.3%-29.6%] vs 11.3% [95% CI, 11.0%-11.7%]; *P* < .001) (Table 2). Compared with individuals with other common food allergies, those with sesame allergy had significantly higher estimated rates of food protein–induced enterocolitis (4.4% [95% CI, 2.4%-7.9%] vs 1.8% [95% CI, 1.4%-2.2%]; *P* = .004) and eosinophilic esophagitis (3.6% [95% CI, 1.4%-8.7%] vs 0.5% [95% CI, 0.4%-0.8%]; *P* < .001).

### Family History

Data on parental history of atopy are presented in Table 2. Among individuals with sesame allergy, 41.0% (95% CI, 31.4%-51.4%) reported a parental history of any food allergy; however, these data were based on self-report irrespective of clinical diagnosis or reaction history. Similarly, a parental history of self-reported allergic rhinitis was reported in 52.5% (95% CI, 42.9%-61.9%). Individuals with sesame allergy were more likely than those with other food allergies to report a parental history of reported asthma (41.0% [95% CI, 31.4%-51.4%] vs 26.6% [95% CI, 24.8%-28.3%]; *P* = .002), eczema (29.9% [95% CI, 22.3%-38.7%] vs 19.7 [95% CI, 18.2%-21.3%]; *P* = .007), allergic rhinitis (52.5% [95% CI, 42.9% vs 61.9%] vs 47.5% [95% CI, 45.4%-49.7%]; *P* = .33), and food allergy (50.4% [95% CI, 40.3%-60.4%] vs 42.4% [95% CI, 40.1%-44.3%]; *P* = .12). Individuals with sesame allergy were also significantly more likely to report a parental history of asthma or eczema compared with those with allergies to 1 or more of the top 8 food allergens (26.6% [95% CI, 24.8%-28.3%] and 19.7% [95% CI, 18.2%-21.3%], respectively; *P* < .001).

### Sesame Allergy Symptoms

Individuals with sesame allergy exhibited gastrointestinal tract symptoms less often than individuals with 1 of the top 8 food allergens (Table 3). For example, 8.3% (95% CI, 4.8%-13.9%) of individuals with sesame allergy reported vomiting as an allergic reaction symptom, compared with 33.4% (95% CI, 31.6%-35.3%) of those with other top 8 allergies. Among the different symptoms, individuals with sesame allergy exhibited hives significantly more often than individuals who are allergic to 1 of the top 8 food allergens (71.6% [95% CI, 63.3%-78.7%] vs 57.9% [95% CI, 56.0%-59.8%]; *P* < .001). No differences among the frequency of cardiovascular symptoms were exhibited between sesame allergy and 1 of the top 8 food allergens.

**Table 3.** Sesame Allergy Reaction Symptoms

Symptom Reported	Population-Weighted Frequency, % (95% CI)		
	Convincing Allergy to Top 8 Allergens (n = 6942)	Convincing Sesame Allergy During Most Severe Reaction to Sesame (n = 251)	Physician-Confirmed, Convincing IgE-Mediated Sesame Allergy Reporting Each Symptom During Most Severe Reaction to Sesame (n = 117)
Skin or oral mucosal tissue			
Hives	57.9 (56.0-59.8)	71.6 (63.3-78.7)	69.5 (58.2-78.8)
Itching	59.3 (57.4-61.2)	54.9 (46.1-63.5)	57.9 (46.1-68.9)
Rash	45.0 (43.1-46.8)	30.3 (23.2-38.5)	39.1 (28.4-51.0)
Swelling	26.7 (25.1-28.4)	17.9 (11.7-26.3)	18.6 (10.9-30.0)
Lip or tongue swelling	32.7 (31.0-34.5)	26.1 (17.8-36.6)	28.6 (19.2-40.4)
Difficulty swallowing	35.5 (33.7-37.3)	20.5 (14.7-28.0)	27.5 (18.1-39.4)
Hoarse voice	17.4 (16.0-18.8)	15.5 (9.9-23.4)	18.6 (10.9-29.9)
Itchy mouth	31.7 (30.0-33.5)	19.2 (13.8-26.2)	25.8 (17.3-36.6)
Throat tightening	31.4 (29.7-33.1)	18.6 (12.6-26.6)	16.3 (9.5-26.7)
Mouth or throat tingling	21.9 (20.3-23.5)	9.7 (6.0-15.2)	13.8 (7.2-24.8)
Respiratory tract			
Chest tightening	22.6 (21.1-24.2)	14.1 (9.4-20.6)	15.6 (8.5-26.9)
Nasal congestion	19.5 (18.0-21.1)	13.6 (8.3-21.5)	15.8 (9.0-26.2)
Repetitive cough	15.8 (14.4-17.3)	7.1 (4.1-11.9)	8.0 (4.1-14.8)
Trouble breathing	29.0 (27.3-30.8)	22.6 (15.9-31.1)	23.1 (14.7-34.4)
Wheezing	21.8 (20.3-23.3)	7.4 (4.4-12.0)	8.5 (4.1-16.8)
Gastrointestinal tract			
Belly pain	36.8 (34.9-38.8)	12.8 (8.3-19.3)	18.3 (10.6-29.8)
Cramps	34.1 (32.2-36.0)	16.6 (10.7-24.9)	18.2 (10.7-29.2)
Diarrhea	35.5 (33.6-37.4)	11.2 (7.2-17.1)	13.3 (7.4-22.7)
Nausea	35.5 (33.7-37.4)	18.6 (12.3-27.1)	17.2 (10.2-27.6)
Vomiting	33.4 (31.6-35.3)	8.3 (4.8-13.9)	8.2 (3.8-17.0)
Cardiovascular			
Chest pain	9.7 (8.7-10.9)	11.5 (7.1-18.0)	14.6 (7.8-25.6)
Rapid heart rate	16.7 (15.4-18.1)	15.6 (9.8-23.9)	13.9 (7.4-24.5)
Fainting or dizziness	16.7 (15.3-18.2)	13.1 (8.5-19.9)	14.8 (8.0-25.7)
Low blood pressure	5.3 (4.5-6.2)	6.8 (2.9-14.8)	4.2 (1.7-10.1)

### Sesame Allergy Characteristics

Information regarding sesame allergy characteristics pooled across children and adults are displayed in Table 4, whereas separate age-stratified estimates are provided in the eTable in the Supplement. A severe reaction to sesame (as indicated by multiple organ system involvement) was reported by 37.2% (95% CI, 29.2%-45.9%) of those with convincing sesame allergy. Severe reactions meeting a more restricted set of symptom-report criteria consistent with other previous work^13^ (ie, sesame-induced wheeze, fainting or dizziness, and/or low blood pressure) were reported by 23.6% (95% CI, 16.9%-32.0%) of individuals with sesame allergy. Most individuals with sesame allergy reported additional food allergies (81.6%; 95% CI, 71.0%-88.9%) that also met our convincing symptom-report criteria, with peanut allergy (46.9%; 95% CI, 38.2%-55.8%) being the most common comorbid food allergy. More than three-quarters (76.7%; 95% CI, 66.4%-84.6%) had an allergy to 1 or more of the top 8 food allergens. A current epinephrine prescription was reported by 62.2% (95% CI, 53.4%-70.4%) of those with sesame allergy. Almost two-thirds (64.6%; 95% CI, 55.0%-73.1%) of individuals with sesame allergy reported having visited the emergency department at least once in their lifetime owing to a food-allergic reaction, although because of time constraints the survey did not assess whether the visit was specifically for treatment of a sesame-allergic reaction. However, among individuals with convincing sesame allergy and no other convincing food allergies, 36.4% (95% CI, 17.3%-61.0%) reported an emergency department visit during their lifetime. More than half (58.7%; 95% CI, 50.1%-66.8%) of individuals with a sesame allergy did not report a physician diagnosis of their allergy. Of those who did have a convincing, physician-confirmed allergy, 73.3% (95% CI, 62.8%-81.7%) were diagnosed using skin prick tests, 37.4% (95% CI, 27.4%-48.6%) via sesame-specific IgE blood tests, and 13.6% (95% CI, 8.2%-21.6%) by oral food challenge. In contrast, patients with a physician-diagnosed sesame allergy who did not report a history of convincing reaction symptoms were more frequently diagnosed via serum IgE testing (52.8%; 95% CI, 36.4%-68.7%), followed by skin prick tests (43.1%; 95% CI, 28.4%-59.2%) and oral food challenge (7.8%; 95% CI, 3.4%-16.9%).

**Table 4.** Sesame Allergy Characteristic

Variable	Population-Weighted Frequency, % (95% CI)		
	Individuals With Convincing Sesame Allergy	Individuals With Physician-Diagnosed, Convincing IgE-Mediated Sesame Allergy	Individuals With Physician-Diagnosed, Sesame Allergy Without Convincing Symptom Report
Severe sesame allergic reaction			
Stringent reaction symptoms occurring within multiple organ systems	37.2 (29.2-45.9)	43.1 (32.4-54.5)	0
Wheeze, fainting or dizziness, and/or low BP only	23.6 (16.9-32.0)	25.9 (17.1-37.2)	0
Physician diagnosed	41.3 (33.2-49.9)	100	100
Adult-onset (among adults only)	25.7 (18.1-35.1)	26.3 (151.0-41.7)	21.0 (11.7-34.7)
Multiple convincing food allergies	81.6 (71.0-88.9)	85.7 (74.9-92.3)	59.6 (32.0-82.2)
Current epinephrine prescription	62.2 (53.4-70.4)	74.2 (63.2-82.8)	77.7 (59.3-89.3)
Food allergy–related ED visits			
≥1 Lifetime	64.6 (55.0-73.1)	69.0 (56.8-79.1)	44.2 (24.0-66.5)
≥1 In the past year	31.8 (24.3-40.5)	31.4 (22.3-42.1)	24.6 (12.4-43.0)
Comorbid allergy			
Peanut	46.9 (38.2-55.8)	51.6 (40.1-63.0)	37.1 (21.4-56.2)
Tree nut	34.8 (27.2-43.3)	36.1 (25.8-47.8)	17.7 (10.5-28.3)
Milk	20.1 (14.8-26.7)	22.2 (14.3-33.0)	11.9 (6.5-20.6)
Shellfish	27.5 (20.7-35.6)	23.5 (14.9-34.9)	9.2 (4.1-19.6)
Egg	24.9 (18.0-33.4)	21.1 (13.3-31.9)	7.1 (3.4-14.3)
Fin fish	21.1 (14.6-29.4)	13.7 (7.7-23.0)	6.0 (2.3-15.0)
Wheat	18.9 (13.6-25.6)	25.1 (16.5-36.3)	5.6 (1.8-16.0)
Soy	26.6 (20.0-34.3)	25.9 (17.2-37.0)	2.3 (1.0-5.3)
Sesame allergy diagnosis			
Skin prick or scratch test	58.3 (47.6-68.3)	73.3 (62.8-81.7)	43.1 (28.4-59.2)
Blood test	29.7 (21.5-39.5)	37.4 (27.4-48.6)	52.8 (36.4-68.7)
OFC	10.8 (6.5-17.3)	13.6 (8.2-21.6)	7.8 (3.4-16.9)
Used to treat a sesame-allergic reaction			
EAI	33.7 (26.3-42.0)	43.3 (32.3-55.1)	9.5 (4.6-18.5)
Antihistamines	49.8 (40.9-58.7)	56.9 (45.3-67.9)	49.9 (33.3-66.5)
Asthma inhaler	9.0 (5.7-14.1)	14.3 (8.1-24.1)	6.0 (2.7-12.8)
Corticosteroids	12.8 (8.3-19.2)	12.5 (7.0-21.3)	5.7 (2.1-14.6)

Abbreviations: BP, blood pressure; EAI, epinephrine autoinjector; ED, emergency department; OFC, oral food challenge.

With respect to treatment of sesame allergy reactions, 33.7% (95% CI, 26.3%-42.0%) reported use of an epinephrine autoinjector at least once in their lifetime, 49.8% (95% CI, 40.9%-58.7%) reported antihistamine use, 9.0% (95% CI, 5.7%-14.1%) reported asthma inhaler use, and 12.8% (95% CI, 8.3%-19.2%) used corticosteroids to treat a sesame-allergic reaction.

## Discussion

This study is the first, to our knowledge, to comprehensively characterize the population-level burden of sesame allergy among US children and adults, concluding that an estimated 0.23% of the US population has a current sesame allergy with a history of convincing IgE-mediated symptoms. An additional 0.11% of the US population reported a current physician-diagnosed sesame allergy without a corresponding history of stringent reaction symptoms. Combined, these estimates suggest that more than 0.34% of the US population, or 1.1 million children and adults, are likely to be directly affected by a current sesame allergy. Overall, a total of 0.49% of the US population, or more than 1.5 million children and adults, may have a current sesame allergy, indicating a greater perceived burden of sesame allergy than previously acknowledged.

Age-specific sesame allergy prevalence estimates indicate that sesame allergy affects children and adults of all ages, with comparable rates exhibited among both groups. These estimates extend previous US survey research published in 2010,^8^ which came to similar conclusions but relied on a much smaller sample of individuals reporting sesame allergy for prevalence estimation (ie, 3 children and 10 adults vs 206 children and 273 adults in the present study). Similarly, a 2010 population-based Canadian prevalence study^13^ reported probable sesame allergy prevalence rates of 0.23% for children and 0.05% for adults based on reaction symptoms and clinical testing. However, like the aforementioned US prevalence survey, findings from this Canadian study^13^ were limited by the small sample of individuals reporting sesame allergy (5 children and 4 adults). Nevertheless, as in the present study, the reported proportions of respondents reporting severe reactions in the population-based Canadian sample were comparable among patients with sesame allergy and those with other, more prevalent allergies (eg, peanut, tree nut, and shellfish).^13^

Our data extend previous work indicating that allergic reactions to sesame can be severe^15,16^ by demonstrating that sesame allergy also results in substantial use of health care services among US children and adults. For example, the present data indicate that nearly 2 in 3 individuals with sesame allergy (with or without comorbid allergies) report having received food allergy treatment in the emergency department compared with approximately 1 in 3 individuals allergic to sesame alone. Furthermore, more than 1 in 3 children and adults with sesame allergy experienced severe allergic reaction symptoms to sesame as defined by multiple stringent symptoms affecting multiple organ systems, whereas approximately 1 in 4 children and adults with sesame allergy experienced wheeze, hypotension, or fainting or dizziness on sesame ingestion. With respect to epinephrine autoinjector use, approximately 1 in 3 individuals reported having previously treated a sesame allergic reaction with an epinephrine autoinjector. This rate is higher than the fewer than 10% of patients with sesame allergy reported by a recent clinic-based regional Canadian study^17^ but comparable to estimates obtained via a prior population-based telephone survey.^13^ At the same time, the present data suggest that fewer than 2 in 3 US children and adults with sesame allergy have a current prescription for an epinephrine autoinjector, indicating that sesame allergy management practices remain suboptimal. Together, the present data indicate a substantial economic, psychosocial, and physical health burden experienced by individuals with sesame allergy living in North America.

To date, few studies have examined the population-level burden of sesame allergy, with data collected from 2008 through 2010 estimating pediatric prevalence rates to be 0.2% in US children^6^ and 0.1% in US adults.^8^ Data from other countries, and particularly those where sesame-containing foods are widely consumed (eg, Israel^16^ and Saudi Arabia^18^), indicate that sesame is a leading cause of food-induced anaphylaxis,^16,18^ with correspondingly high prevalence rates (eg, 0.8% among Australian infants diagnosed via confirmatory oral food challenge).^19^ In Canada, annual rates of accidental allergen exposure are comparable between children with sesame and peanut allergies.^17^ Rates and sources of accidental sesame exposure among US children were not assessed by the present study, but this remains an important area for future research.

Currently, the Food Allergen Labeling and Consumer Protection Act of 2004 (FALCPA)^11^ mandates labeling of the top 8 allergenic foods or food groups (peanut, milk, shellfish, tree nuts, egg, wheat, soy, and fin fish) along with proteins derived from them. Protection from exposure to these allergens in flavoring, spices, colors, and processing aids is provided to consumers through required labeling,^20^ but this protection is not in place for sesame in the United States. Although Australia, Canada, Europe, and New Zealand require labeling,^7^ the United States currently has no requirement to clearly identify sesame as an ingredient on food packaging, despite comparable rates of sesame allergy.^13^ Furthermore, based on additional data collected in the present survey, multiple food allergies explicitly addressed by FALCPA appear to be substantially less prevalent than sesame allergy. For example, data from this same survey indicate that convincing pine nut and macadamia nut allergies currently affect 0.10% (95% CI, 0.07%-0.14%) and 0.008% (95% CI, 0.004%-0.018%) of US children and adults, respectively, using identical convincing IgE-mediated symptom report criteria. This issue of sesame allergen labeling may be increasingly important in coming years, given that sesame consumption appears to be increasing domestically and abroad.^21^

With respect to the natural history of sesame allergy, the reported median ages of first reaction to sesame were comparable to those reported previously in Canada among children and adults.^13^ Our data suggest that approximately 1 in 4 adults with sesame allergy developed their allergy as adults.^2^ These findings are consistent with previous work in Israel suggesting that sesame allergy is relatively persistent,^22^ with 20%^23^ to 30%^24^ of infants with allergies estimated to outgrow their allergy during childhood. However, further prospective studies are needed to more rigorously characterize the natural history of sesame allergy in the US context.

In light of long-standing concerns about the overestimation of food allergy prevalence via survey-based approaches, previous survey-based food allergy prevalence studies^6,8^ (including this one) have attempted to reduce false-positive findings by requiring that respondents report specific allergic reaction symptoms indicative of an IgE-mediated reaction. However, such symptom report–based definitions risk misclassifying individuals with true food allergies who have only experienced mild symptoms or who have not yet tried the food owing to deliberate sesame avoidance after clinical testing. Such misclassification may be particularly likely for patients with sesame allergy, owing to the fact that sesame allergy appears to co-occur at high rates with other allergies that tend to develop earlier in life. For example, these data suggest that nearly half of those with a convincing sesame allergy are allergic to peanuts, one-third are allergic to tree nuts, one-quarter are allergic to eggs, and 1 in 5 have a comorbid milk allergy. In our study, only 51.3 (95% CI, 41.5%-61.0%) of individuals reporting a physician-diagnosed sesame allergy fulfilled our strict symptom report criteria for a convincing IgE-mediated allergy, although more than two-thirds reported previous reaction symptoms after exposure to sesame. Another important consideration is that, in contrast to some other allergens, sesame seeds are small and frequently sprinkled on foods unground, which may reduce their allergenicity compared with such allergenic seeds consumed in ground format. This consideration may help explain the relatively large proportion of physician-diagnosed sesame allergy accompanied by mild symptoms observed in the present study. Together, these data appear to indicate that the true population-level burden of sesame allergy is likely to exceed the estimated 0.23% of the US population who report a sesame allergy and a convincing IgE-mediated reaction history.

### Limitations

Although strengths of our study include its large, nationally representative sample and survey-based design, which permits estimation of the prevalence of clinically confirmed and unconfirmed food allergies alike, recall bias may influence symptom report of sesame-specific symptoms, particularly for nonrecent reactions. Similarly, use of health care services and other allergy characteristics were only assessed via self-report and parent proxy. However, it is worth noting that in our study, sesame allergy characteristics (eg, symptoms, severity) did not differ substantially between individuals with sesame allergy with and without a physician diagnosis, supporting the idea that a substantial proportion of IgE-mediated sesame allergy in the United States is not physician diagnosed, including many severe sesame allergies. Nevertheless, by relying on survey-based assessments and not incorporating confirmatory clinical allergy testing, it remains difficult to ascertain how many of the more than 1.5 million US children and adults (0.49% of the US population) who perceive themselves to be allergic to sesame truly have a current IgE-mediated sesame allergy. The fact that self-reported or perceived food allergy is associated with psychosocial impairment and decreased quality of life owing to the challenges of allergen avoidance underlines the importance of confirmatory allergy testing irrespective of test outcome.^25,26,27,28,29^ In addition, the relatively low rates of physician diagnosis observed among individuals with convincing IgE-mediated sesame allergy would seem to indicate potential missed opportunities for patient counseling and education, as well as prescription of potentially life-saving medication.

## Conclusions

Overall, these findings suggest that IgE-mediated sesame allergy is likely to affect at least 1 million children and adults in the United States, with an estimated 0.49% of the population reporting a current sesame allergy, 0.34% reporting convincing IgE-mediated sesame reaction symptoms of a current sesame allergy or a current physician diagnosis of sesame allergy, and 0.23% having a convincing sesame allergy. In addition, many experience severe reactions and substantial food allergy–related use of health care services. We believe these data, which demonstrate a substantial and likely growing burden of sesame allergy in the United States, provide valuable context to physicians, policy makers, and other key stakeholders in their efforts to evaluate and reduce the public health burden of sesame allergy.

## Notes

Supplement. **eFigure 1.** List of Allergic Reaction Symptoms Highlighting Stringent Symptoms Indicative of Convincing Food Allergy **eFigure 2.** Convincing, Physician-Confirmed, and Severe Food Allergy Categorization Flowchart **eTable.** Sesame Allergy Characteristic for Children and Adults, Population-Weighted Frequency (95% CI)
