# Supplementary material for: Prevalence and Severity of Sesame Allergy in the United States
Source: JAMA Netw Open. 2019 Aug 2;2(8):e199144. doi: 10.1001/jamanetworkopen.2019.9144 (PMC6681546; doi:10.1001/jamanetworkopen.2019.9144)
Supplement: Supplement. — eFigure 1. List of Allergic Reaction Symptoms Highlighting Stringent Symptoms Indicative of Convincing Food Allergy eFigure 2. Convincing, Physician-Confirmed, and Severe Food Allergy Categorization Flowchart eTable. Sesame Allergy Characteristic for Children and Adults, Population-Weighted Frequency (95% CI) [file jamanetwopen-2-e199144-s001.pdf]

Supplementary Online Content

Warren CM, Chadha A, Sicherer SH, Jiang J, Gupta RS. Prevalence and severity of sesame allergy in the United States. *JAMA Netw Open.* 2019;2(8):e199144. doi:10.1001/jamanetworkopen.2019.9144

eFigure 1. List of Allergic Reaction Symptoms Highlighting Stringent Symptoms Indicative of Convincing Food Allergy

eFigure 2. Convincing, Physician-Confirmed, and Severe Food Allergy Categorization Flowchart

eTable. Sesame Allergy Characteristic for Children and Adults, Population-Weighted Frequency (95% CI)

This supplementary material has been provided by the authors to give readers additional information about their work.

© 2019 Warren CM et al. *JAMA Network Open.*

eFigure 1. List of Allergic Reaction Symptoms Highlighting Stringent Symptoms Indicative of Convincing Food Allergy

□ Skin/Oral Mucosa Symptoms
- □ ***Hives***
- □ Itching
- □ Rash
- □ ***Swelling*** (except lip/tongue swelling)
- □ ***Lip/tongue swelling***
- □ ***Difficulty swallowing***
- □ Hoarse voice
- □ Itchy mouth
- □ ***Throat tightening***
- □ Mouth or throat tingling
- □ Other: ___________________

□ Respiratory Symptoms
- □ ***Chest tightening***
- □ Nasal congestion
- □ Repetitive cough
- □ ***Trouble breathing***
- □ ***Wheezing***
- □ Other: ___________________

□ Gastrointestinal (GI) Symptoms
- □ Belly pain
- □ Cramps
- □ Diarrhea
- □ Nausea
- □ ***Vomiting***
- □ Other: ___________________

□ Cardiovascular/Heart Symptoms
- □ ***Chest pain***
- □ ***Rapid heart rate***
- □ ***Fainting, dizziness, or feeling light headed***
- □ ***Low blood pressure***
- □ Other: __________________

□ Other Symptoms
- □ Anxiety
- □ Feeling of impending doom
- □ Headache
- □ Other: ___________________

All symptoms listed are offered as answer choices in the survey. Symptoms in bold italics comprised our expert panel's stringent symptom list.
A "convincing" food allergy required the report of at least one stringent symptom during the participant's most severe reaction to a given food.

Figure Legend: The above figure has been published in-

1. Gupta RS, Warren CM, Smith BM, et al. The public health impact of parent-reported childhood food allergies in the United States. *Pediatrics.* 2018;142(6).
2. Gupta RS, Warren CM, Smith BM, et al. Prevalence and severity of food allergies among US adults. *JAMA Network Open.* 2019;2(1):e185630.

© 2019 Warren CM et al. *JAMA Network Open.*

eFigure 2. Convincing, Physician-Confirmed, and Severe Food Allergy Categorization Flowchart

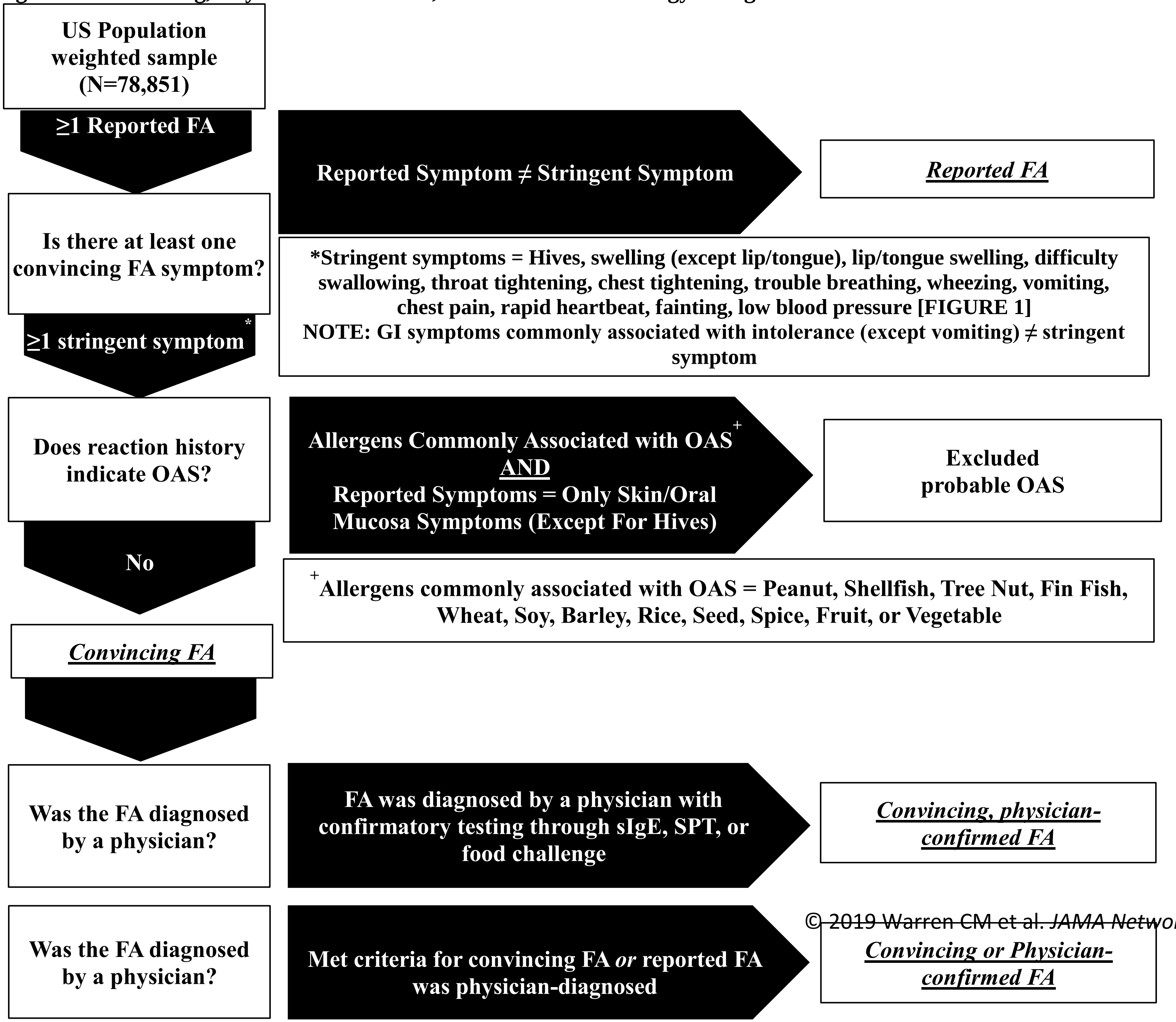

© 2019 Warren CM et al. *JAMA Network Open.*

Figure Legend: Similar figures have been published in-

1. Gupta RS, Warren CM, Smith BM, et al. The public health impact of parent-reported childhood food allergies in the United States. *Pediatrics.* 2018;142(6).
2. Gupta RS, Warren CM, Smith BM, et al. Prevalence and severity of food allergies among US adults. *JAMA Network Open.* 2019;2(1):e185630.

© 2019 Warren CM et al. *JAMA Network Open.*

eTable. Sesame Allergy Characteristic for Children and Adults, Population-Weighted Frequency (95% CI)

Variable:	**% of individuals with convincing sesame allergy**	**% of children with convincing sesame allergy**	**% of adults with convincing sesame allergy**	**% of individuals with physician-diagnosed, convincing IgE-mediated sesame allergy**	**% of children with physician-diagnosed, convincing IgE-mediated sesame allergy**	**% of adults with physician-diagnosed, convincing IgE-mediated sesame allergy**	**% of individuals with physician-diagnosed, sesame allergy without convincing symptom report**	**% of children with physician-diagnosed, sesame allergy without convincing symptom report**	**% of adults with physician-diagnosed, sesame allergy without convincing symptom report**
Severe Sesame Allergic Reaction (i.e. Stringent reaction symptoms occurring within multiple organ systems)	37.2 (29.2-45.9)	27.2 (17.5-39.7)	39.7 (30.3-49.9)	43.1 (32.4-54.5)	29.9 (17.9-45.3)	48.1 (34.3-62.3)	0	0	0
Severe Sesame Allergic Reaction (i.e. Wheeze, Fainting/Dizziness and/or Low BP only)	23.6 (16.9-32.0)	19.2 (10.9-31.7)	24.7 (17.2-34.2)	25.9 (17.1-37.2)	14.7 (7.7-26.1)	30.2 (18.7-44.8)	0	0	0
Physician Diagnosed	41.3 (33.2-49.9)	55.5 (41.6-68.6)	37.7 (28.7-47.6)	100	100	100	100	100	100
Adult-onset Sesame Allergy (among adults only)	25.7 (18.1-35.1)	N/A	25.7 (18.1-35.1)	26.3 (15.0-41.7)	N/A	26.3 (15.0-41.7)	21.0 (11.7-34.7)	N/A	34.9 (21.3-51.4)

© 2019 Warren CM et al. *JAMA Network Open.*

Multiple convincing food allergies	81.6 (71.0-88.9)	86.4 (69.2-94.7)	80.3 (67.5-88.9)	85.7 (74.9-92.3)	83.2 (57.3-94.8)	86.6 (74.1-93.6)	59.6 (32.0-82.2)	42.7 (11.1-81.6)	72.4 (52.1-86.3)
Variable:	**% of individuals with convincing sesame allergy**	**% of children with convincing sesame allergy**	**% of adults with convincing sesame allergy**	**% of individuals with physician-diagnosed, convincing IgE-mediated sesame allergy**	**% of children with physician-diagnosed, convincing IgE-mediated sesame allergy**	**% of adults with physician-diagnosed, convincing IgE-mediated sesame allergy**	**% of individuals with physician-diagnosed, sesame allergy without convincing symptom report**	**% of children with physician-diagnosed, sesame allergy without convincing symptom report**	**% of adults with physician-diagnosed, sesame allergy without convincing symptom report**
One or more Lifetime FA-related ED Visits	64.6 (55.0-73.1)	58.2 (44.3-70.9)	66.2 (54.6-76.2)	69.0 (56.8-79.1)	59.5 (40.3-76.1)	72.6 (57.0-84.2)	44.2 (24.0-66.5)	26.4 (7.6-60.7)	57.8 (37.0-76.1)
One or more FA-related ED Visits in the Past year	31.8 (24.3-40.5)	33.0 (22.2-46.0)	31.5 (23.1-41.5)	31.4 (22.3-42.1)	30.0 (17.7-46.0)	31.9 (20.8-45.6)	24.6 (12.4-43.0)	15.2 (3.9-43.9)	31.8 (16.5-52.4)
Comorbid Peanut Allergy	46.9 (38.2-55.8)	55.2 (41.1-68.5)	44.8 (34.9-55.0)	51.6 (40.1-63.0)	47.7 (30.4-65.6)	50.8 (36.7-64.7)	37.1 (21.4-56.2)	53.3 (21.8-82.3)	26.4 (15.6-41.1)
Comorbid Tree Nut Allergy	34.8 (27.2-43.3)	43.9 (31.0-57.7)	32.5 (23.9-42.4)	36.1 (25.8-47.8)	37.0 (22.1-55.0)	35.2 (22.8-50.0)	17.7 (10.5-28.3)	14.5 (5.6-32.3)	19.8 (10.9-33.3)
Comorbid Milk Allergy	20.1 (14.8-26.7)	22.7 (14.2-34.3)	19.4 (13.4-27.3)	22.2 (14.3-33.0)	16.8 (7.1-34.7)	16.1 (8.0-29.7)	11.9 (6.5-20.6)	14.5 (5.6-32.3)	12.9 (6.5-23.8)
Comorbid Shellfish Allergy	27.5 (20.7-35.6)	31.0 (19.1-46.2)	26.7 (19.0-36.0)	23.5 (14.9-34.9)	21.5 (10.2-39.8)	14.6 (6.9-28.4)	9.2 (4.1-19.6)	10.4 (3.4-27.8)	10.0 (3.6-25.2)
Comorbid Egg Allergy	24.9 (18.0-33.4)	26.3 (16.0-40.1)	24.5 (16.9-34.1)	21.1 (13.3-31.9)	25.6 (13.2-43.7)	12.7 (5.6-26.1)	7.1 (3.4-14.3)	8.7 (2.8-24.2)	6.0 (2.1-15.9)

© 2019 Warren CM et al. *JAMA Network Open.*

Comorbid Fin Fish Allergy	21.1 (14.6-29.4)	23.0 (12.9-37.7)	20.6 (13.7-29.7)	13.7 (7.7-23.0)	14.2 (6.5-28.3)	10.7 (4.5-23.3)	6.0 (2.3-15.0)	6.0 (1.2-24.5)	6.0 (1.7-19.0)
Comorbid Wheat Allergy	18.9 (13.6-25.6)	30.8 (19.5-45.0)	15.9)10.4-23.4)	25.1 (16.5-36.3)	36.9 (20.6-56.9)	17.8 (9.3-31.3)	5.6 (1.8-16.0)	5.9 (1.2-24.4)	5.3 (1.1-22.7)
Comorbid Soy Allergy	26.6 (20.0-34.3)	25.6 (16.2-38.1)	26.8 (19.2-36.1)	25.9 (17.2-37.0)	22.6 (11.2-40.2)	20.1 (10.9-34.1)	2.3 (1.0-5.3)	2.1 (.6-6.9)	2.4 (.8-7.3)
Sesame Allergy diagnosed by Skin Prick/Scratch Test	58.3 (47.6-68.3)	73.8 (59.0-84.7)	53.5 (40.7-65.8)	73.3 (62.8-81.7)	80.9 (66.9-89.9)	70.5 (56.9-81.2)	43.1 (28.4-59.2)	32.7 (13.4-60.4)	50.1 (34.6-65.5)
Sesame Allergy diagnosed by Blood Test	29.7 (21.5-39.5)	41.3 (26.3-58.1)	26.1 (16.9-38.1)	37.4 (27.4-48.6)	45.3 (28.6-63.1)	34.4 (22.6-48.5)	52.8 (36.4-68.7)	80.0 (58.4-91.9)	34.7 (21.2-51.3)
Variable:	**% of individuals with convincing sesame allergy**	**% of children with convincing sesame allergy**	**% of adults with convincing sesame allergy**	**% of individuals with physician-diagnosed, convincing IgE-mediated sesame allergy**	**% of children with physician-diagnosed, convincing IgE-mediated sesame allergy**	**% of adults with physician-diagnosed, convincing IgE-mediated sesame allergy**	**% of individuals with physician-diagnosed, sesame allergy without convincing symptom report**	**% of children with physician-diagnosed, sesame allergy without convincing symptom report**	**% of adults with physician-diagnosed, sesame allergy without convincing symptom report**
Used EAI to treat a sesame-allergic reaction	33.7 (26.3-42.0)	30.2 (19.3-44.1)	34.5 (25.9-44.3)	43.3 (32.3-55.1)	42.1 (25.2-61.0)	43.8 (30.4-58.2)	9.5 (4.6-18.5)	4.4 (1.4-13.0)	12.9 (5.8-26.2)
Used Antihistamines to treat a sesame-allergic reaction	49.8 (40.9-58.7)	41.3 (28.6-55.3)	52.0 (41.6-62.1)	56.9 (45.3-67.9)	46.2 (29.0-64.4)	61.0 (46.4-73.8)	49.9 (33.3-66.5)	51.0 (20.0-81.2)	49.2 (33.8-64.7)
Used Asthma Inhaler to treat	9.0 (5.7-14.1)	11.2 (6.3-19.1)	8.5 (4.7-14.8)	14.3 (8.1-24.1)	16.3 (8.3-29.6)	13.5 (6.2-27.2)	6.0 (2.7-12.8)	12.4 (4.2-31.6)	1.8 (0 .4-7.0)

© 2019 Warren CM et al. *JAMA Network Open.*

a sesame-allergic reaction									
Used Steroids to treat a sesame-allergic reaction	12.8 (8.3-19.2)	17.4 (8.0-33.9)	11.6 (6.9-18.7)	12.5 (7.0-21.3)	21.7 (9.1-43.6)	9.0 (4.3-17.9)	5.7 (2.1-14.6)	6.7 (1.1-30.7)	5.0 (1.6-14.4)

© 2019 Warren CM et al. *JAMA Network Open.*
